# The Adulteration of Milk in Bristol

**Published:** 1909-03

**Authors:** Edward Russell

**Affiliations:** Analyst for the City and County of Bristol


### THE ADULTERATION OF MILK IN BRISTOL.

BY

EDWARD RUSSELL, B.Sc., F.I.C.,

*Analyst for the City and County of Bristol.*

---

THE composition of milk may be regarded as varying between 12 to 13 parts of total solid matter, and the fat of this amount between 3.3 and 4.3, and the non-fatty solids between 8.5 and 9 parts. The constituents of the non-fatty solids are not taken into account in judging the purity of milk samples under the Food and Drug Acts, and the percentages of lactose, proteid and ash are not therefore usually determined. The first standard suggested by the Society of Public Analysts in 1875 was, fat 2.5 per cent., non-fatty solids 9.0. The solids were determined by drying 5 or 10 cc. in a platinum dish to a constant weight, and then extracting the fat from this portion by means of dry ether; by this means the fat was never completely extracted, and hence a low minimum for fat and a higher for solids were at first fixed. About ten years later, with the introduction of improved processes of analysis, such as the Adams, Schmidt and others, the figures were altered to 2.7 fat, and 8.5 solids other than fat. No figure has ever been fixed for total solid matter. This standard was a possible minimum for the milk of a single cow, but the work of Vieth and Richmond showed that the genuine milk of a mixed herd did not fall below 3 per cent. except in an infinitesimal percentage of abnormal specimens; the Board of Agriculture, therefore, adopted the present standard of fat 3 per cent., non-fatty solids 8.5 per cent. at the beginning of this century. All samples condemned under the Food and Drug Acts fall below this standard. This standard is very much exceeded as regards fat at all times of the year, except in the spring, while as regards solids other than fat the spring is the high period.

It is not usual in genuine milk to find a sample approaching either limit in which the other limit is not greatly exceeded.

The total solids of genuine milk should not be less than 12.0 per cent. When I first undertook analyses of Bristol milk, I was struck by the number of samples which were of bare legal quality, and I was at once led to conclude that it is a practice here amongst skilled adulterators to add the requisite amount of separated milk and water to genuine samples of good quality in order to produce milk which would just pass the analyst. In this they occasionally fail, and hence the high Bristol figures for adulteration. The following are the numbers of the last two years :—

| Examined. | Adulterated. | Per cent. condemned. | No. watered. | No. deficient in fat. |
|---|---|---|---|---|
| 650 | 82 | 12.9 | 18 | 63 |
| 647 | 101 | 15.8 | 36 | 63 |

The increase of 3 per cent. in the adulteration figures is shown to be entirely due to the addition of water, and as some of the samples so condemned contained only 3 or less than 3 per cent. of fat, I have no doubt that many of these are the result of skilful manipulation to produce legal milk which just failed in its object.

The only satisfactory result in recent years in the milk supply of Bristol is the reduction in the use of preservatives. I have never found formaldehyde; and the samples condemned for boric acid, which amounted to twenty, three years ago, during the last two years have only amounted to three.

